# A portable isometric knee extensor strength testing device: test-retest reliability and minimal detectable change scores of the Q-Force ӀӀ in healthy adults

**DOI:** 10.1186/s12891-021-04848-8

**Published:** 2021-11-19

**Authors:** Johanneke Hartog, Sandra Dijkstra, Joke Fleer, Pim van der Harst, Massimo A. Mariani, Lucas H. V. van der Woude

**Affiliations:** 1 Department of Cardiothoracic Surgery, University of Groningen, University Medical Center Groningen, Groningen Groningen, The Netherlands; 2 Department of Health Sciences, University of Groningen, University Medical Center Groningen, Groningen, the Netherlands; 3 Department of Cardiology, University of Groningen, University Medical Center Groningen, Groningen, the Netherlands; 4 Center for Human Movement Sciences, Groningen and Department of Rehabilitation Medicine, University of Groningen, University Medical Center, Groningen, the Netherlands; 5 School of Sport Exercise and Health Sciences, Peter Harrison Centre for Disability Sport, Loughborough University, Loughborough, UK

**Keywords:** Muscle strength, Muscle weakness, Therapeutics, Resistance training, Quadriceps muscle, Muscular atrophy, Rehabilitation, Reliability

## Abstract

**Background:**

Although knee extensors are essential in daily activities (e.g. walking, climbing stairs), knee extensor strength is often not measured in clinical settings. Existing devices to test muscle strength are not always suitable to accurately measure the high forces of this muscle group. Therefore, a device to test muscle strength that is convenient, feasible, reliable, and valid in clinical settings is required. This study evaluated the reliability, responsiveness, and level of discomfort of the newly developed Q-Force ӀӀ (i.e. a portable device to measure isometric knee extensor strength) in healthy middle-aged and elderly adults.

**Methods:**

Participants (*n* = 22) conducted two standardized test sessions on the Q-Force ӀӀ (five to ten days apart). Each session consisted of one familiarisation trial followed by three trials of peak isometric knee extension per each leg. Per trial, peak and mean knee extension force (N) and torque (Nm) were measured at 90° flexion. The level of discomfort was determined using a visual analog scale (VAS: 0-100). Intra Class Correlation (ICC, model: two-way mixed with absolute agreement), Standard Error of Measurement (SEM), and minimal detectable change (MDC) were determined. A repeated measures ANOVA was used to determine between-test variation.

**Results:**

Excellent test-retest (ICC > 0.95) and inter-trial (ICC > 0.91) reliability for both legs were shown. No significant differences were found in peak and mean knee forces and torques between test and retest of both legs, indicating good test-retest reliability (*P*-value range: 0.360-0.538; F(1,21) range: 0.4-0.9). The SEM of the peak and mean forces and torques ranged from 28.0 to 30.4 N (6.0-6.8%) and from 9.2 to 10.4 Nm (6.4-7.7%), respectively. The MDC for these outcomes ranged respectively from 77.6 to 84.1 N (16.5-18.8%) and from 25.5 to 28.9 Nm (17.6-21.4%). The level of discomfort was low (median range: 7-10, IQR: 4-18).

**Conclusion:**

The portable Q-Force ӀӀ is a comfortable, responsive, and relatively cheap device with excellent test-retest reliability. This device would be potentially suitable to measure isometric knee extensor strength in clinical settings.

**Supplementary Information:**

The online version contains supplementary material available at 10.1186/s12891-021-04848-8.

## Introduction

Daily activities, such as walking, climbing stairs, and rising from a chair, requires a considerable effort of the knee extensors [1]. Decline in knee extensor strength can cause limitations in normal activities of daily life and may seriously impact functioning, participation, and independency [2, 3]. Loss of skeletal muscle strength occurs with ageing and is related to risk of falls, impaired cognitive functions, decreased quality of life, and increased mortality. In addition, it is commonly seen in non-communicable diseases and after hospitalization [3–9]. As the risks of physical inactivity [10] and the benefits of exercise have been well-established [11], strengthening interventions are increasingly implemented as part of medical treatment (i.e. ‘Exercise is Medicine’). For example, preoperative rehabilitation has been suggested to improve outcomes after cardiac surgery [12]. To evaluate the efficacy of clinical exercise interventions on knee extensor strength in clinical settings, a device to test muscle strength is required that is convenient, feasible, reliable, and valid.

Despite the important role of knee extensor muscles in everyday activities, this muscle group receives limited attention in clinical studies. Existing devices to measure muscle strength are not always suitable to measure this muscle group accurately in clinical settings. Isokinetic dynamometry (e.g. Biodex, Kincom) is considered to be the gold standard to measure peak (isometric and isokinetic) muscle strength using force sensor technology [13, 14]. However, these dynamometers are expensive, relative immobile, and difficult to use in clinical settings. In addition, accessibility of the device may be limited in clinical populations and the device requires an expert observer for reliable test results [15, 16]. In contrast, handheld dynamometers (HHDs, e.g. MicroFET) are portable, relatively inexpensive, and easy to use in clinical settings. They can be used at the bed-site or at home, and observers are easily trained [17]. However, major disadvantages of the HHD include the susceptibility of the uni-dimensional force sensor to position and orientation errors of both participant and observer. The trained therapist would need to standardize the position and orientation of the sensor to the participant’s body segment and, more importantly, stabilize the HHD during the trial. Stabilizing and maintaining the required force direction with the HHD is challenging when the participant is applying maximum (leg) force [16, 18], especially when measuring the high forces of the lower extremities (e.g. knee extensors) up to 500 N [19]. Indeed, reliability coefficients of HHDs for knee extension are lower compared to body joints that are less forceful, such as elbow flexion and shoulder extension [20]. These coefficients were also lower, when compared to isokinetic testing or when knee extensor strength was measured by an observer with a weak or average strength level [20–22]. The strength of the observer to position the sensor adequately thus limits reliable measurements of isometric knee extension strength with an HHD, also in clinical settings [23].

Solutions to stabilize the HHD and portable chair-based strength measurements have been developed to improve the stability and positioning of the force transducer [24–28]. In this situation, the stabilization of the force transducer is less dependent on the tester, and is rather determined by the stiffness and stabilization of the stabilizer or instrumented device. A chair-based device, the Q Force, a successor of the Quadriso Tester [28], was developed specially for the knee extensors by Douma et al. [27]. Compared to an HDD (with or without the use of a stabilizer), the Q Force device has the advantage to generate strength curves, which gives more insight in how fast the force is generated. In addition, the fixed leg brace located at the front of the Q Force stabilizes the force sensor perpendicular to the lower leg, omitting the need to find a stabilization point in a certain space, which is needed when using an HHD with stabilizer. By using different stabilization points, the attachment of the force sensor and the posture of the participant are potentially less standardized, providing possibly a suboptimal force sensing direction, which affect the magnitude of the measured force (N).

**Fig. 1:**
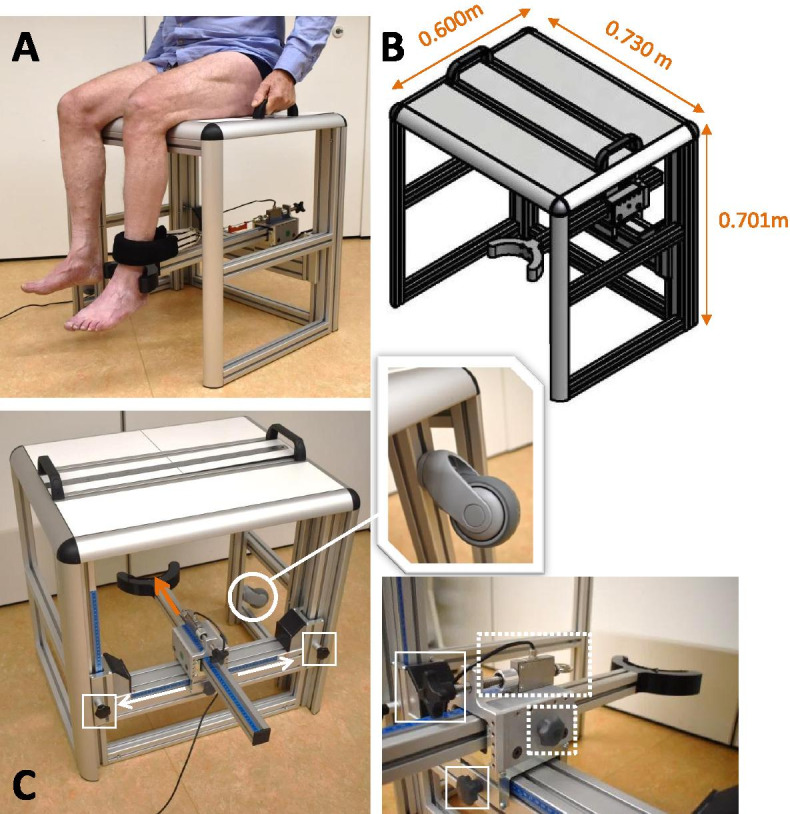
Overview of the Q-Force ӀӀ. **A** Performance of a maximal isometric knee extensor with the Q-Force ӀӀ; **B** Technical graph of the Q force with the dimensions; **C** White arrows (sliding directions of the force transducer) are perpendicular to the direction of produced knee extension force (orange arrow), preventing sliding of the force transducer during a measurement. *Dotted white rectangle:* Force transducer; *White circle*: wheels are attached at the side so that it can be transported; *White squares:* buttons to slide the force transducer upwards, sideward and forward making it possible to adjust the force transducer to the left and right leg and adapt is to different leg lengths of the participant; *Dotted white square:* Button to move the bar, which supports the heel, forward and backward

Recently, a newly instrumented isometric knee extension platform, the Q-Force ӀӀ, was developed, which compares favourably with previous instruments due to the use of stronger and stiffer material. This minimizes bending or deformation of the material, preventing the force sensor of moving relative to the lower leg when high forces are applied to the device. In addition, the force transducer of the Q-Force ӀӀ is located at the back of the chair frame, instead of the front. This additionally prevents anterior-posterior movements of the leg brace and the force sensor connected to it, because the fixation of the force transducer is perpendicular to the direction of the pulling force produced by knee extension onto the sensor (Fig. 1). Compared to the original Q Force, the Q-Force ӀӀ contains a wider seating area and the adjustability of the force transducer is considerably larger, making it suitable for shorter and taller individuals, as well as individuals with higher BMI. The aim of this study was to determine the reliability and responsiveness of the peak knee extensor strength in healthy middle-aged and elderly adults. Furthermore, the level of discomfort when using the Q-Force ӀӀ was evaluated. We hypothesised that the Q-Force ӀӀ has a good reliability, is responsive for change, and participants report a low level of discomfort when using the Q-Force ӀӀ.

## Methods

### Study design

For each participant, two standardized test sessions on the Q-Force ӀӀ were conducted five to ten days apart. These test and retest sessions were planned at the same time of the day. Participants were asked to adhere to their normal lifestyle and to avoid heavy exercises in the period between the two test sessions. During each of the two measurements, participants were asked to perform four voluntary maximal isometric knee extension contractions with each leg with a 2 minute rest period between each of the trials. Within this set of trials, the first one was executed to familiarise the participant with the set-up and test procedure. Using a counterbalanced order (stratified for age [45-55, 55-65, and 65-76 years] and gender [M/F]) it was determined whether the participant started with the left or right leg. All methods were carried out in accordance with the Minimum Standards of Reporting Checklist and the STROBE-guidelines.

### Participants

In this study, 22 healthy middle-aged to elderly adults (gender: 11/11, age: 59.4 ± 8.7 years, BMI: 25.0 ± 3.1 kg/m^2^) volunteered and were included after giving written informed consent. Participants did not have a chronic disease affecting the hands and/or legs, no history of a stroke or cardiovascular disease, and were not diagnosed with osteoporosis in the legs. Furthermore, nobody recently (within 3 months) had any injury to the lower extremities or received medical advice to refrain from leg exercises. The median of days that participants exercised (e.g. walking, cycling, strength training, ball games, or other sports) was 3.5 (IQR: 2-6) days per week with a median of 60 (IQR: 45-90) minutes per day. In 18 participants (82%) the right leg was dominant (i.e. the leg used to kick a ball), while in the remaining four participants (18%) it was the left leg [29]. The study was approved by the Ethics Committee of the Center for Human Movement Sciences, University Medical Center Groningen (UMCG, ECB/2016.11.02_1).

### Device Q-force ӀӀ

**Fig. 2:**
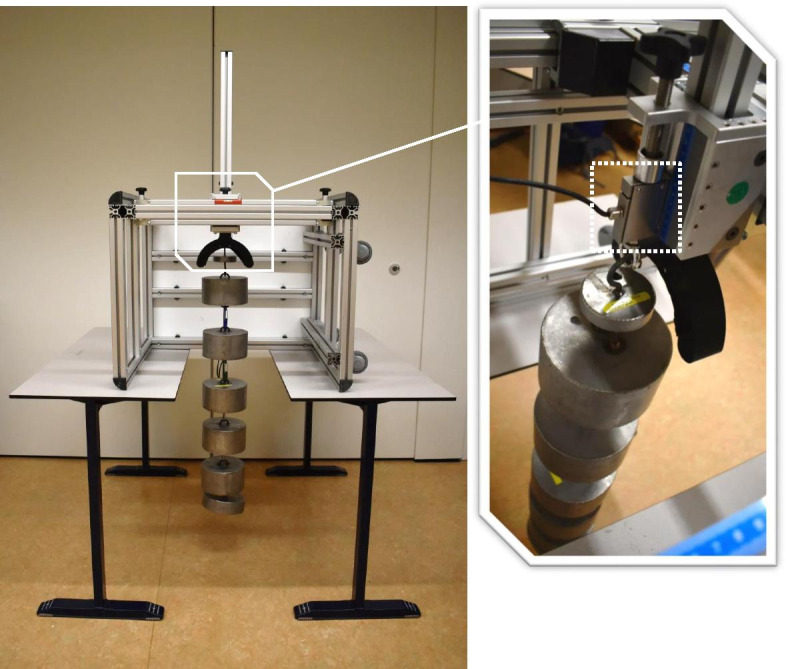
Calibration procedure of the Q-Force ӀӀ. The Q-Force ӀӀ is rotated a quarter turn (90°) positioning the force transducer sensing direction to vertical. A series of reference loads of 1, 10, and 20 kg were statically applied in a stepwise procedure (10-110 kg) onto the force transducer. Dotted white square: Force transducer

The Q-Force ӀӀ is a custom made isometric knee extension force platform (Technical Support, Centre for Human Movement Sciences and Research Support Facility, UMCG, Groningen, the Netherlands) intended for clinical use. Figure 1 shows the dimensions and construction of the Q-Force ӀӀ. The frame is built with 40 × 40 N aluminium profiles (BOIKON B.V., Leek, the Netherlands). There are two handles on the seat, which participants used to stabilize themselves and standardize the test procedure. The chair is instrumented with a uni-dimensional force sensor (load cell KAP-E/2kN, A.S.T. Mess- und Regeltechnik, Dresden, Germany) attached to a crossbar at the back. A leg strap, which is wrapped around the lower leg, is connected to the force sensor with a stainless steel wire and positioned horizontal and perpendicular to the lower leg to determine the generated force (N). The force sensor is connected to a computer using an analogue-digital converter (NIUSB-6001) with a USB connection. The Q-Force 2.0 software (Technical Support, Center for Human Movement Sciences, UMCG, Groningen, the Netherlands) displays the generated Force (100 Hz) on screen during the trial and uses a second order low-pass-recursive-Butterworth filter (10 Hz) for detailed data collection. The device can be adjusted to the side of the leg (left/right), to different leg lengths and body dimensions of participants by shifting the force sensor to the left and right and by shifting the crossbar up and down (Fig. 1). A bar, placed under the load cell and to the front of the chair, supports the heel and helps maintaining a fixed knee angle (90°). Standard settings of the standardized position of each participant and the force transducer are obtained by using the five rulers on the frame of the Q-Force ӀӀ. In order to obtain similar knee and leg orientation towards the force transducer for all participants when repeating the measurement on different occasions and locations. The Q-Force ӀӀ can be easily calibrated when the Q-Force ӀӀ is rotated 90° to the front, so that the sensor direction of the force transducer is positioned vertically, when the Q-Force ӀӀ is placed on two identical tables and the reference loads can be applied statically to the force transducer (Fig. 2). The measured force should be equal to the weight of the reference loads times the gravitational acceleration. Wheels are attached to the right side of the Q-Force ӀӀ to facilitate transportation of the device (Fig. 1C).

### Procedures

Before signing informed consent, participants were asked to fill in the PAR-Q questionnaire [30]. Additional questions were asked to determine any reasons for exclusion and whether participants were engaged in heavy physical activities in the week prior to the first measurement. During the first measurement, baseline parameters, including body weight and length, dominant leg, and lower leg length, were obtained. The length of the lower leg was measured from the inter tibial condylar point to the point lying between the medial and lateral malleolus. The distance was measured in line with the tuberosity of the tibiae on the ventral side of the lower leg. Caffeine consumption and medication use within 2 h prior to the measurement were also noted at each test and retest.

Participants were invited to sit in the centre (marked by a line) of the Q-Force ӀӀ chair, with their knees bent and the popliteal fossa against the seat edge. Keeping their lower legs hanging down without tension and at an angle of 90°. The bar to support the heel was placed behind the heel and the bottom side of the leg strap was placed 0.03 m above the inter-malleolar point, which is located at the ventral side of the lower leg between the medial and lateral malleolus (Fig. 1A). The load cell was placed horizontal and perpendicular to the lower leg, which was controlled with a line level (Fig. 1A). The stainless steel wire, connecting the load cell with the leg strap, was placed in line with de midline of the heel bar, making the leg strap and load cell horizontally aligned. The load cell was moved backwards until a preload of 20 N to 30 N was created. The preload was measured during 6 seconds before each trial. During these 6 seconds the participants were instructed to sit up straight (or ‘to straighten their back’), with hands on the handles to fixate themselves, and relaxed legs. Participants were instructed to perform four trials of peak isometric knee extensions of 6 seconds each, following a standardized procedure: ‘build up strength gradually to peak strength in three seconds and hold the peak strength for the remaining three seconds’. The researcher coached the participant by saying ‘build up slowly’ in the first 3 s and ‘come on, come on, come on!’ in the final 3 seconds. Participants were carefully instructed and observed to maintain their position during a trial, to keep their hands on the handles, sit up straight, and not lean backwards or forwards. No oral or visual performance feedback was given to participants. Immediately after a trial, participants were asked whether the contraction performed was maximal and filled out a 0.10 m Visual Analogue Scale (VAS) about the level of discomfort during the trial. A VAS score of 0 m corresponded to “no discomfort” and 0.10 m corresponded to “very severe discomfort”. Spontaneously reported feelings of discomfort during or after the trial were also noted. The measurements were conducted by two trained researchers. Prior to each session the Q-Force ӀӀ was calibrated following a fixed protocol and in a range of 10 to 110 kg, using weights of 1, 10, and 20 kg (Fig. 2).

### Outcomes and data-analyses

**Fig. 3:**
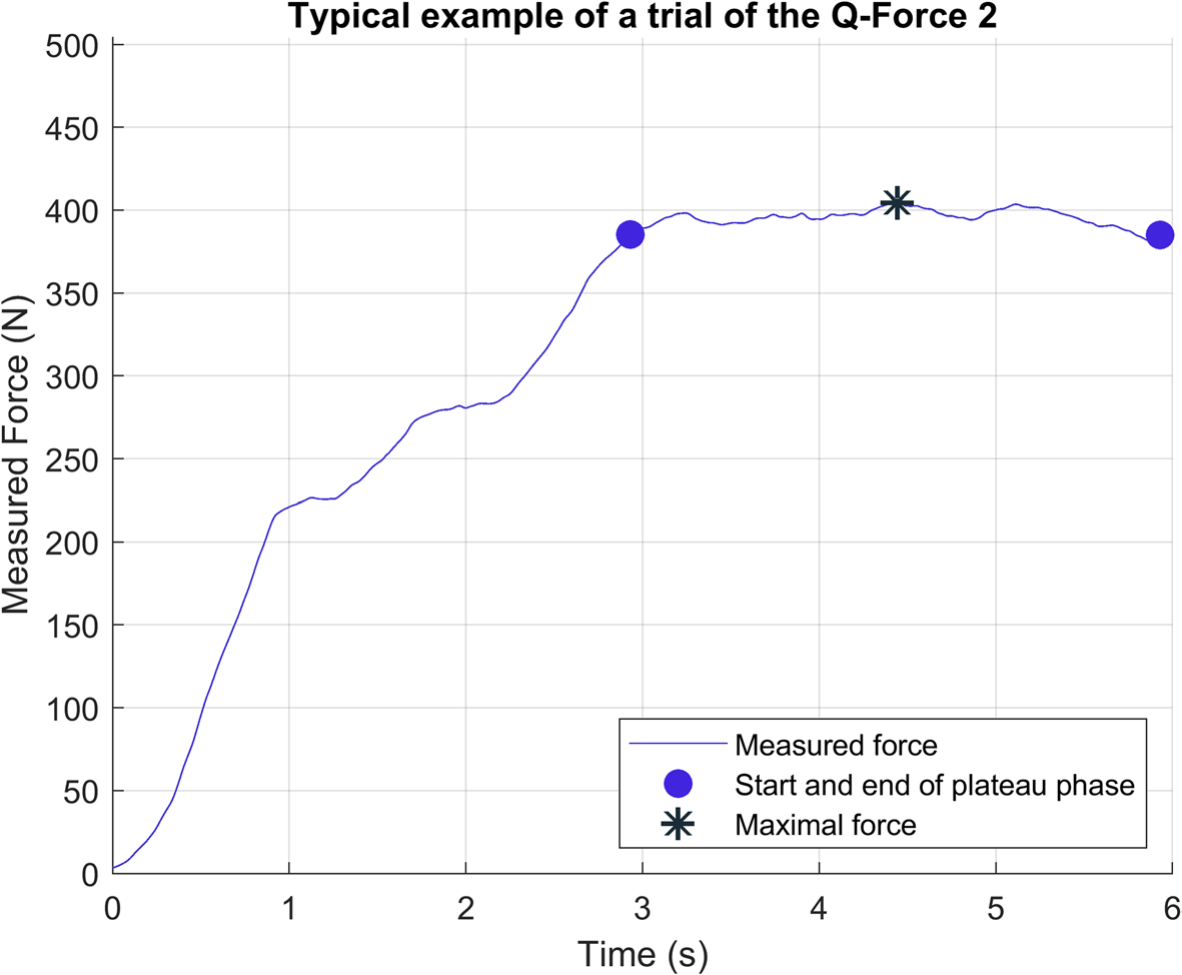
Typical example of a trial of the Q-Force ӀӀ

Per leg, the main outcomes were the measured knee extension peak and mean force (N), the calculated peak and mean torque (Nm), and the level of discomfort (VAS: 0-100). The knee extension torque during a trial was calculated from the product of the measured force (N) and the arm (m) of the force to the rotation point of the knee point (Eq. 1). The arm was defined as the distance from the force sensor to the ventral inter tibial condylar point. The ventral inter tibial condylar point was thus considered as the rotation point of the knee joint. The peak force (F-peak) and torque (T-peak) were defined as the maximum value during a trial. The average of the force (F-mean) and torque (T-mean) of the plateau phase were also derived. The plateau phase was defined as the three consecutive seconds of the trial where the participants generated the highest torque on average, which was determined by selecting the highest 3-s moving average (Fig. 3).

1 $$Torque\ (Nm)=\left( Measured\ force\ (N)- preload\ (N)\right)\ x\ arm\ (m)$$

### Sample size

Based on the reliability study of the Q force, a sample size of 24 participants would be needed to detect an ICC greater than 0.80 with a confidence interval width of 0.3 [27, 31].

### Statistical analyses

Baseline variables and trial results were summarized using descriptive statistics. The Intra Class Correlation (ICC, model: two-way mixed with absolute agreement) was used to determine the test-retest (Type: Average measures) and inter-trial reliability (Type: Single measures) [32]. Values less than 0.50 were classified as poor reliability, values between 0.50 and 0.75 as moderate reliability, values between 0.75 and 0.90 as good reliability, and values greater than 0.90 were classified as excellent reliability [32]. A repeated measures ANOVA, followed by Bonferroni post-hoc tests, was used to determine differences in means between the test and the retest (within factor measurement), the three trials (within factor trial), and both legs (within factor leg) for F-peak, T-peak, F-mean, and T-mean as dependent variables. Degrees of freedom were adjusted according to Greenhouse-Geisser when sphericity was violated. The Wilcoxon signed-rank test was used when there was no normal distribution. The standard error of measurement (SEM) was estimated as an absolute index for the test-retest reliability using Eq. 2, in which SD was the standard deviation [33]. Equation 3 was used to calculate the minimal detectable change (MDC, at 95% confidence interval) as a measure of responsiveness [33]. The MDC represents the minimum difference that can be considered as a real change in muscle force. The ICC, SEM, and MDC were determined using the average of the three trials. Level of significance was set at *P* < 0.05 and statistical tests were all two-sided.

2 $$SEM= SD\ast \surd \left(1- ICC\right)$$

3 $$MDC= SEM\ x\ 1.96\ x\surd 2$$

## Results

All participants successfully conducted the test and retest measurements. A typical trial is shown in Fig. 3. The median number of days between the test and the retest was seven (IQR: 7.0-7.0).

**Fig. 4:**
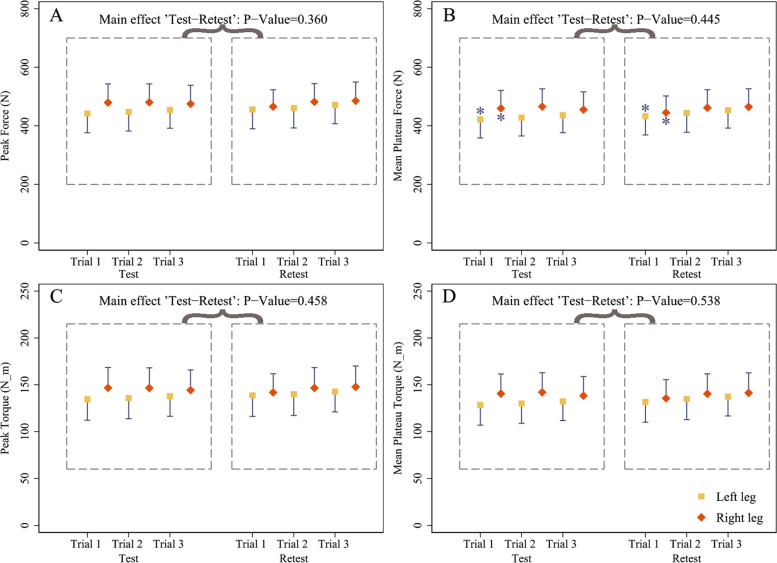
Mean with 95% confidence interval (plotted to one side) of the three trials at the Q-Force ӀӀ of the test and retest. **A** Peak force; **B** The average force of the plateau phase; **C** Peak Torque; **D** The average torque of the plateau phase. *Trial 1 is significantly different from trial 2 and 3 in Bonferroni post hoc tests (*P*-value< 0.05)

For the test-retest reliability, all ICCs (averaged over the trials) were larger than 0.95 indicating an excellent reliability (Table 1). Furthermore, no significant differences were found between the test and the retest (Fig. 4, F(1,21) = 0.9, 0.6, 0.6, and 0.4 for respectively F-peak, T-peak, F-mean, and T-mean). The SEM of the peak and mean force and torque ranged from 28.0 to 30.4 N (6.0-6.8%) and from 9.2 to 10.4 Nm (6.4-7.7%), respectively (Table 1). The MDC for these outcomes ranged from 77.6 to 84.1 N (16.5-18.8%) and from 25.5 to 28.9 Nm (17.6-21.4%), respectively (Table 1).

**Table 1:** Test-retest reliability measures for isometric knee extensor force (N) and Torque (Nm) (*n* = 22)

Force (N)	Leg	Test^**a**^ (mean ± SD)	Retest^**a**^ (mean ± SD)	ICC* (95% CI)	SEM (%)^b^	MDC (%)^**c**^
**Peak**	Left	448.1 ± 144.5	462.8 ± 148.3	0.978 (0.946-0.991)	30.4 (6.7)	84.1 (18.5)
	Right	478.4 ± 141.7	477.8 ± 136.5	0.978 (0.948-0.991)	28.5 (6.0)	79.0 (16.5)
**Mean**	Left	429.1 ± 138.8	443.6 ± 143.1	0.977 (0.944-0.991)	29.6 (6.8)	82.0 (18.8)
	Right	460.4 ± 135.9	457.3 ± 134.1	0.978 (0.947-0.991)	28.0 (6.1)	77.6 (16.9)
**Torque (Nm)**						
**Peak**	Left	136.1 ± 49.3	140.5 ± 50.0	0.977 (0.946-0.991)	10.4 (7.5)	28.9 (20.9)
	Right	145.9 ± 48.5	145.4 ± 47.9	0.981 (0.954-0.992)	9.3 (6.4)	25.7 (17.6)
**Mean**	Left	130.3 ± 47.1	134.7 ± 48.1	0.977 (0.944-0.990)	10.2 (7.7)	28.3 (21.4)
	Right	140.3 ± 46.4	139.1 ± 46.7	0.980 (0.952-0.992)	9.2 (6.6)	25.5 (18.2)

^a^ Mean of 3 trials; ^b^% SEM expressed as a percentage of the average strength measure of the test and retest; ^c^% MDC expressed as a percentage of the average strength measure of the test and retest. *All *P*-values < 0.001; *CI* Confidence Interval, *ICC* Intra Class Correlation, *MDC* Minimal Detectable Change, *SD* Standard deviation, *SEM* Standard Error of Measurement

The reliability between the trials was also excellent with ICCs larger than 0.91 (Additional file 1: Table S1). The repeated measures ANOVA showed no significant main effect of ‘Trial’ for T-peak (*P*-value = 0.103, F(1.5,31.0) = 2.6). For the three remaining variables this effect was significant (F-peak: *P*-value = 0.039, F(1.5,32.5) = 3.9; F-mean: *P*-value = 0.010, F(1.4,30.3) = 6.3; T-mean: *P*-value = 0.032, F(1.4,29.2) = 4.5). Bonferroni post-hoc tests showed that only for F-mean, trial 1 was significantly different from trials 2 and 3 (*P*-value< 0.05, Fig. 4).

The T-mean of the right leg was higher compared to the left leg (*P*-value = 0.046, F(1,21) = 4.5), but for the other outcomes the level of significance was not reached (F-peak: *P*-value = 0.067, F(1,21) = 3.7; T-peak: *P*-value = 0.060, F(1,21) = 4.2; F-mean: *P*-value = 0.052, F(1,21) = 3.9). The interaction effects (measurement*trial, measurement*leg, trial*leg, measurement*trial*leg) were not significant (*P*-value> 0.05).

The median discomfort VAS-scores ranged from 7 to 10 (IQR: 4-18, scale 0-100, with higher scores indicating a higher level of discomfort) for both legs, trials, and measurements. One participant reported discomfort VAS-scores above 60 for all trials of the left leg of the test session. The main reason given for these high scores was the duration of sitting without back support. This participant, however, indicated that he could continue the test as long as needed. Furthermore, this participant reported lower VAS-scores during the retest session, since the duration of this session was shorter. Other participants reported an uncomfortable sensation of the edge of the seating against the popliteal fossa (*n* = 4), the pressure of the leg strap (*n* = 3), knee complaints (n = 3), uncomfortable sensations of the muscle contracting (*n* = 2), and one participant reported numbness of the feet (*n* = 1). None of the reported feelings of discomfort led to discontinuation of the research protocol, and any unpleasant feeling occurred during a trial only and diminished easily after the trial was finished.

## Discussion

In this study, the reliability, responsiveness, and level of discomfort of the Q-Force ӀӀ were evaluated. Results showed an excellent test-retest and inter-trial reliability for both legs. No significant differences were found between the test and retest in peak and mean of the measured forces and torques of both legs, indicating good test-retest reliability. Moreover, the SEM and MDC for peak and mean knee extension torque ranged respectively from 9.2 to 10.4 Nm (6.4-7.7%) and 25.5 to 28.9 Nm (17.6-21.4%). Finally, participants reported low levels of discomfort during the Q- Force ӀӀ trials.

The reliability of the Q-Force ӀӀ was excellent with ICCs above 0.95 (test-retest) and above 0.91 (inter-trial), which was higher compared to the ICCs in HHD studies (0.50-0.90) [21, 23, 34, 35] and consistent with the ICCs determined in HHDs that employed a stabilizer (0.87-0.96) [21,24-26], portable chair-based strength devices (0.89-0.96) [27, 28], and isokinetic dynamometers (0.93-0.98, Table 2) [14, 36, 37].

**Table 2:** Reported reliability measures (ICC, SEM, and MDC) of different knee extensor strength devices

Author	Year	Sample	Age Mean ± SD	n	Device (stabilizer)	TBS	Knee angle	Leg	ICC	Torque (Nm) Mean ± SD	SEM (%)^a^	MDC (%)^**b**^
**HHD**												
Bohannon [21]	2013	Healthy adults	26.0 ± 2.4	8	MicroFET 2	7 days	90°	NR	0.50-0.77^d^	UtC	UtC	UtC
Roebroeck [23]	1998	IOKD	35 (13-77)	30	CAHN-DE	1 h	25°	NR	NR	44^e^	6.9 (15.7)	19.2 (43.6)
Bohannon [34]^c^	1993	Acute hospitalized	NR	21	Ametek	NR	NR	Left	0.95	34.8 ± 26.5	5.9 (17.1)	13.4 (38.5)
								Right	0.84	36.8 ± 30.0	12.0 (32.6)	27.6 (75.1)
Roy [35]	2004	Hip fracture	79 ± 7	16	Lafayette	1-2 days	90°	Un-fractured	0.90	45.8 ± 13.2	4.2 (9.2)	11.6 (25.4)
**Fixed HHD**												
Bohannon [21]	2013	Healthy adults	26.0 ± 2.4	8	MicroFET 2 (belt)	7 days	90°	NR	0.87-0.92^d^	UtC	UtC	UtC
Jackson [24]	2017	Athletes	34.3 ± 3.5	15	MicroFET 2 (StabD)	15 min	90°	Left & Right	0.93	80.9 ± 22.4	5.9 (7.3)	16.4 (20.3)
Katoh [26]	2014	Healthy Elderly	65 to 79	186	цTas F-1 (belt)	30 s	90°	Dominant	0.96	9.6 ± 3.0	0.6 (6.3)	1.7 (17.4)
Katoh [25]	2009	Healthy adults	21.9 ± 1.5	37	цTas MF-01 or F-1 (belt)	7 days	90°	Dominant (right)	0.91	14.3 ± 4.6	1.4 (9.7)	3.8 (26.8)
**Portable chair-based strength devices**												
Verkerke [28]	2003	Healthy adults	28.1 ± 6.5	20	Quadriso-tester	1 day	90°	Left	0.94	184.5 ± 48.6	11.9 (6.5)	33.0 (17.9)
								Right	0.92	188.8 ± 47.6	13.4 (7.1)	37.3 (19.8)
Douma [27]	2016	Healthy Elderly	81.9 ± 4.9	42	Q Force	3-8 days	70°	Left	0.89	66.9 ± 28.2	9.4 (14.0)	25.9 (38.8)
								Right	0.96	70.5 ± 32.3	6.5 (9.2)	17.9 (25.4)
**Isokinetic dynamometer**												
Maffiuletti [14]	2007	Healthy adults	30 ± 5	30	Con-Trex	7 days	60°	Dominant	0.98	217.8 ± 59.8	7.8 (3.6)	21.6 (9.9)
Carvalho [36]	2013	Healthy adults	23 ± 3	24	REV9000	7 days	60°	Left	0.93	235.5 ± 44.7	11.8 (4.8)	32.8 (13.3)
								Right	0.96	253.0 ± 52.7	10.5 (4.2)	29.2 (11.5)
Flansbjer [37]	2010	Persons with late effects of Polio	63 ± 6.4	30	Biodex 3 PRO	7 days	90°	Less-affected	0.98	137.7 ± 59.1	8.4 (4.7)	24.5 (17.8)^f^

*ICC* Intra Class Correlation, *IOKD* Isolated orthopaedic knee disorders, *MDC* Minimal Detectable Change, *NR* Not reported, *SEM* Standard Error of Measurement, *TBS* Time between sessions, *UtC* Unable to calculate; 0° = full extension. ^a^Estimated using equation: SD * wortel (1-ICC); ^b^Estimated using equation: SEM*1.96*√(2); ^c^Information extracted from Bohannon et al., 2010 [34]; ^d^Range ICC from male and female testers; ^e^Estimated from Fig. 2 of Roebroeck et al., 1998 [23]; ^f^Mean of upper and lower limit

The measured torques of the Q-Force ӀӀ ranged from 130.3 to 145.9 Nm. Due to the use of strong and stiff materials for build, the Q-Force ӀӀ seems better equipped to resist forces above the estimated upper limit of HHDs of 85 Nm [23]. In contrast to the reliability study of the first version of the Q Force [27], we found no significant differences or interaction effects between the test and the retest for the Q-Force ӀӀ. This suggests that systematic errors (e.g. learning effects, fatigue) can be significantly attenuated in a measurement schedule of three trials after a familiarisation trial, thereby improving the test-retest reliability.

Next to systematic errors, random errors may always be present due to biological variability, instrumentation, and conducting errors by the subject or tester [33]. Therefore, the SEM is an important reliability measure, which represents these measurement errors in absolute values. Table 2 shows the estimated SEM of a subset of comparable studies. The results of the Q-Force ӀӀ (SEM: 6.4-7.7%) were better compared to the HHDs studies (SEM: 9.2-32.6%) [21, 23, 34, 35], comparable to the studies using HHDs with a stabilizer (SEM: 6.3-9.7%) [21, 24–26] and portable chair-based strength devices (SEM: 6.5-14.0%) [27, 28], and slightly higher than studies with isokinetic dynamometers (SEM: 3.6-4.8%, Table 2) [14, 36, 37].

In this study, the MDC indicates whether a difference in the test re-test outcomes is due to a real change in isometric knee extension strength. The Q-Force ӀӀ was responsive to change with an estimated MDC ranging from 25.5 to 28.9 Nm (17.6-21.4%) for the peak and mean torque. The responsiveness of the Q-Force ӀӀ was smaller compared to the HHDs studies (MDC: 25.4-75.1%) [21, 23, 34, 35], comparable to the studies using HHDs with a stabilizer (17.4-26.8%) [21, 24–26] and portable chair-based strength devices (17.9-38.8%) [27, 28], and higher compared to isokinetic dynamometers studies (9.9-17.8%, Table 2) [14, 36, 37].

Cautious interpretation is warranted when comparing the results of this study with other studies. First, it should be noted that the torques of the HHD studies mentioned in Table 2, were estimated by using the mean lever arm to the knee joint of the present study (0.30 m), since only the measured force (N) and not the torque (Nm) was reported (Table 2). Although it requires an extra measurement to determine the lever arm, it is important to report the torque, since the torque is independent of the position of the leg. Furthermore, it is the torque that contributes to the rotation of a body segment about a joint making it fundamental for all movements during normal daily activities. Secondly, the range of reported measured torques varied from 9.6 to 253.0 Nm. Next to variation between devices, such a wide range could be explained by variation in age, underlying illness, and trainability within the study group [14, 21, 23–25, 27, 28, 34, 36, 37]. In addition, different methods (e.g. different knee angles [14, 23, 27, 36]) and procedures (e.g. time between sessions [24, 26, 28]) could contribute to this range in measured torques. Therefore, the percentage SEM and MDC (%) were preferred to make comparisons between different studies, which takes into account this wide range and standard deviation of the absolute values.

The measured knee extensor strength of the right leg was slightly stronger compared to the left leg. Possibly because the right leg was dominant in 82% of the participants. Also, the reliability and responsive measures were slightly better for the right leg compared to the left leg. Although, dominance is a factor that could influence outcomes, this is not consistently reported (Table 2). The choice to measure the left, right, dominant, non-dominant, affected and/or non-affected side is dependent on the research question and population. We were interested in the reliability of both left and right leg, because we anticipate using the Q-Force ӀӀ in patients undergoing coronary bypass grafting. In this group of patients, a vein from the upper leg is often used to bypass the coronary stenosis and this may affect the choice of leg to be measured (in our hospital generally a vein of the right leg is used). For other study populations, for example in studies of the aetiology of injuries in soccer players, the dominant vs. non-dominant leg might be of more interest. We present our analyses of the dominant vs. non-dominant leg in Additional file 2. These results showed the same course as the analyses for the left vs. right leg with even smaller SEM and MDC values (resp. 4.8-5.4% and 13.4-15.0%), indeed approximating the reliability and responsiveness levels of studies on isokinetic dynamometers [14, 36, 37]. However, researchers should be aware of a measurement*leg interaction effect, which assumes a learning effect in the non-dominant leg.

When measuring muscle strength, the test results should reflect the maximum force that could voluntarily be achieved. Feelings of discomfort may inhibit the maximum voluntary contraction. Since in this study the reported scores of discomfort were low, we assume minimal interference of discomfort on the utilization of Q-Force ӀӀ. To make the device even more comfortable, a small layer of soft coating is suggested for the seating surface and edge.

The Q-Force ӀӀ can only measure isometric knee extensor strength in 90°. This position is preferred, because ideally the lower leg is in a vertical position, while the force sensor is in the horizontal position, perpendicular to the lower leg. Both knee extensor force and torque can then fairly easy be determined (the latter with using the measured torque arm), without the need to adjust the gravitational and other biomechanical forces acting on the lower leg [38]. Although the knee extensor forces may produce higher forces in a larger knee angle [39], in our opinion standardized accurate measurements of knee extension force in the context of change, i.e. the progression of the force over time, is more important in clinical studies.

Due to the COVID-19 lockdown, the sample size was limited to 22 participants instead of 24, however this sample size was more than sufficient to detect an ICC greater than 0.95 with a confidence interval width of ~ 0.05 (Table 1) [31]. Furthermore, the sample size was comparable to other studies [14, 23, 24, 28, 35, 36]. A strength of this study was that the measurements were conducted by trained testers using a standardized protocol. In addition, to minimize the learning effect over the two sessions a practice session was introduced to familiarise participants with the measurements.

This study included healthy middle-aged and elderly adults since age-related decrease in muscle mass and/or strength (i.e. sarcopenia) begins at about 45 years of age [40]. Furthermore, this age group is more vulnerable to diseases compared to younger persons and may therefore be of interest in clinical studies [41]. However, patients might have different strength levels and strength control compared to healthy peers, possibly resulting in different SEMs and MDCs. As mentioned earlier, these measures play an important role in the interpretation of the progress in knee extension strength during a training or rehabilitation programme. Future research should therefore measure the SEMs and MDCs in different patient groups. In addition, a comparison between the Q-Force ӀӀ and an isokinetic dynamometer would add valuable information about the validity of the Q-Force ӀӀ.

## Practical applications

The Q- Force ӀӀ has the advantage over hand-held dynamometry as it can reliably measure higher forces and it is more responsive to change. If the purpose or research question allows, it is recommended to measure the dominant leg. This achieves higher reliability and responsiveness compared to the HHD with stabilizer. When this is not possible, the Q force ӀӀ still has advantages over HHD with stabilizer, despite having similar reliability and responsiveness. First, the Q-Force ӀӀ is designed to enforce a standardized measurement independent of the space in which knee extension strength is measured and thus the fixation can be set the same over repeated measurements. The reliability of using the Q-force ӀӀ in clinical practice is therefore expected to be more reliable compared to an HHD with stabilizer, where the fixation options are different and thus less standardized. Another advantage is the possibility to generate strength curves, which gives more insight in the rate of torque development and the coordination of the contraction. For one outcome (F-mean) trial 1 was significantly different compared to trials 2 and 3. The findings of this study suggest that performing three measurement trials after a familiarisation trial is appropriate for gaining reliable test-retest measurements. In addition, it is recommended to report the torque as outcome. The Q-Force ӀӀ is easy to transport and relatively cheap (our ‘in-house’ production costs were ~ €3.250) compared to isokinetic devices. The Q-Force ӀӀ is not only an affordable purchase, but also low-cost in maintenance. For example, it is not difficult to calibrate the Q-Force ӀӀ. The device can therefore be used in various (hospitalized) clinical populations, such as patients undergoing cardiac surgery. For example, it can be used to identify sarcopenia, since low muscle strength is the primary parameter in diagnosing this common muscle disorder [42]. Besides, the Q-Force ӀӀ is also suitable for measuring the isometric knee extensor strength of (elite) athletes, because it can reliably measure high forces.

## Conclusions

The portable Q-Force ӀӀ is a comfortable, responsive, relatively cheap, and feasible device in its use among healthy adults. It is easy in use and maintenance, making it a potentially suitable instrument to measure knee extensor strength in a clinical setting. This study established an excellent test-retest reliability when using the Q-Force ӀӀ for measuring isometric knee extensor strength in middle-aged and older healthy adults. In future, the (intra- and inter-observer) reliability and validity of the Q-Force ӀӀ should be evaluated among different patient groups.

## Supplementary Information

**Additional file 1: Table S1**. The Intra Class Correlation between the three trials for the test and retest test for the peak torque and the mean torque of the plateau phase for both legs.

**Additional file 2:.** Results of the test-retest reliability of the dominant and non-dominant leg.

## Data Availability

The dataset generated and analysed during the current study is not publicly available, because no informed consent for publication of the dataset was obtained from the participants. The dataset is available from the corresponding author on reasonable request.

## Abbreviations

F-mean: The average of the force of the plateau phase
F-peak: Peak force, defined as the maximum value during a trial
HHD: Handheld dynamometer
ICC: Intra class correlation
IQR: Interquartile range
MDC: Minimal Detectable Change
SEM: Standard Error of Measurement
T-mean: The average of the torque of the plateau phase
T-peak: Peak torque, defined as the maximum value during a trial
UMCG: University Medical Centre Groningen
VAS: Visual Analogue Scale
