# Supplementary material for: A portable isometric knee extensor strength testing device: test-retest reliability and minimal detectable change scores of the Q-Force ӀӀ in healthy adults
Source: BMC Musculoskelet Disord. 2021 Nov 19;22:966. doi: 10.1186/s12891-021-04848-8 (PMC8602994; doi:10.1186/s12891-021-04848-8)
Supplement: Supplementary file 1 — Additional file 1: Table S1. The Intra Class Correlation between the three trials for the test and retest test for the peak torque and the mean torque of the plateau phase for both legs. [file 12891_2021_4848_MOESM1_ESM.docx]

## Additional file 1

Table S1: The Intra Class Correlation between the trial 1 & 2, 1 & 3, and 2 &3 for the test and re-test test for the peak force and torque and the mean force and torque of the plateau phase for both legs.

|  | **Peak force (N)** | | | | **Mean force (N)** | | | |
| --- | --- | --- | --- | --- | --- | --- | --- | --- |
|  | **Left leg** | | **Right leg** | | **Left leg** | | **Right leg** | |
| **Test** | **Trial 2** | **Trial 3** | **Trial 2** | **Trial 3** | **Trial 2** | **Trial 3** | **Trial 2** | **Trial 3** |
| **Trial 1** | 0.977 | 0.968 | 0.981 | 0.974 | 0.966 | 0.956 | 0.981 | 0.969 |
| **Trial 2** | X | 0.978 | X | 0.976 | X | 0.976 | X | 0.971 |
| **Re-test** |  |  |  |  |  |  |  |  |
| **Trial 1** | 0.981 | 0.970 | 0.968 | 0.939 | 0.985 | 0.968 | 0.962 | 0.935 |
| **Trial 2** | X | 0.984 | X | 0.987 | X | 0.984 | X | 0.991 |
|  | **Peak Torque (Nm)** | | | | **Mean Torque (Nm)** | | | |
|  | **Left leg** | | **Right leg** | | **Left leg** | | **Right leg** | |
| **Test** | **Trial 2** | **Trial 3** | **Trial 2** | **Trial 3** | **Trial 2** | **Trial 3** | **Trial 2** | **Trial 3** |
| **Trial 1** | 0.981 | 0.972 | 0.983 | 0.977 | 0.971 | 0.961 | 0.983 | 0.973 |
| **Trial 2** | X | 0.982 | X | 0.979 | X | 0.980 | X | 0.974 |
| **Re-test** |  |  |  |  |  |  |  |  |
| **Trial 1** | 0.984 | 0.975 | 0.976 | 0.952 | 0.988 | 0.973 | 0.972 | 0.952 |
| **Trial 2** | X | 0.985 | X | 0.990 | X | 0.986 | X | 0.993 |

For all values P-value < 0.001

Table S2: The Intra Class Correlation between the three trials for the test and re-test test for the peak force and torque and the mean force and torque of the plateau phase for both legs.

| **Force (N)** | **Leg** | **Test**  **Trial 1-3** | **Retest**  **Trail 1-3** |
| --- | --- | --- | --- |
| **Peak** | Left | 0.975 | 0.978 |
|  | Right | 0.977 | 0.965 |
| **Mean** | Left | 0.966 | 0.979 |
|  | Right | 0.974 | 0.963 |
| **Torque (Nm)** |  |  |  |
| **Peak** | Left | 0.978 | 0.982 |
|  | Right | 0.980 | 0.973 |
| **Mean** | Left | 0.971 | 0.982 |
|  | Right | 0.977 | 0.973 |

For all values P-value < 0.001
