# Supplementary material for: A portable isometric knee extensor strength testing device: test-retest reliability and minimal detectable change scores of the Q-Force ӀӀ in healthy adults
Source: BMC Musculoskelet Disord. 2021 Nov 19;22:966. doi: 10.1186/s12891-021-04848-8 (PMC8602994; doi:10.1186/s12891-021-04848-8)
Supplement: Supplementary file 2 — Additional file 2:. Results of the test-retest reliability of the dominant and non-dominant leg. [file 12891_2021_4848_MOESM2_ESM.docx]

## Additional file 2

### Results of the test-retest reliability of the dominant and non-dominant leg

For the test-retest reliability, all ICCs (averaged over the trials) were larger than 0.95 indicating an excellent relative reliability (Table S2.1). Furthermore, no significant absolute differences were found between the test and the retest (Figure S2.1, F(1,21)=0.9, 0.6, 0.6, and 0.4 for respectively F-peak , T-peak, F-mean, and T-mean). The SEM of the peak and mean force and torque ranged from 20.5 to 21.2 N (4.4-4.8%) and from 6.8 to 10.0 Nm (4.8-7.2%), respectively (Table S2.1). The MDC for these outcomes ranged from 57.5 to 58.9 N (12.3-13.2%) and from 18.7 to 27.7 Nm (13.4-27.7%), respectively (Table S2.1).

Table S2.1: Test-retest reliability measures for isometric knee extensor force (N) and Torque (Nm) for the dominant and non-dominant leg (n=22).

| **Force (N)** | **Leg** | **Test^1^**  **(mean±SD)** | **Retest^1^**  **(mean±SD)** | **ICC***  **(95% CI)** | **SEM (%)**^2^ | **MDC (%)^3^** |
| --- | --- | --- | --- | --- | --- | --- |
| **Peak** | D | 481.1±149.6 | 474.9±143.5 | 0.979 (0.950-0.991) | 21.2 (4.4) | 58.9 (12.3) |
|  | ND | 445.4±135.7 | 465.8±141.8 | 0.977 (0.931-0.991) | 21.0 (4.6) | 58.3 (12.8) |
| **Mean** | D | 462.1±143.0 | 455.1±139.7 | 0.979 (0.951-0.991) | 20.5 (4.5) | 56.8 (12.4) |
|  | ND | 427.4±131.0 | 445.8±137.9 | 0.976 (0.933-0.990) | 20.8 (4.8) | 57.7 (13.2) |
| **Torque (Nm)** |  |  |  |  |  |  |
| **Peak** | D | 146.2±50.5 | 144.0±49.0 | 0.980 (0.953-0.992) | 7.0 (4.8) | 19.5 (13.4) |
|  | ND | 135.8±47.2 | 141.9±49.1 | 0.978 (0.940-0.991) | 7.1 (5.1) | 19.8 (14.3) |
| **Mean** | D | 140.4±48.1 | 138.0±47.5 | 0.980 (0.954-0.992) | 6.8 (4.9) | 18.7 (13.5) |
|  | ND | 130.3±45.4 | 135.8±47.5 | 0.976 (0.939-0.990) | 7.2 (5.4) | 19.9 (15.0) |

^1^ Mean of 3 trials; ^2^ SEM expressed in percentage of average strength; ^3^ MDC expressed in percentage of average strength. *All P-values < 0.001; CI: Confidence Interval; D: Dominant; ICC: Intra Class Correlation; MDC: Minimal Detectable Change; ND: Non-dominant; SD: Standard deviation; SEM: Standard Error of Measurement.

The reliability between the trials was also excellent with ICCs larger than 0.93 (Table S2.2 & S2.3). The repeated measures ANOVA showed no significant main effect of ‘Trial’ for T-peak (P-value=0.103, F(1.5,31.0)=2.6). For the three remaining variables this effect was significant (F-peak: P-value=0.039, F(1.5,32.5)=3.9; F-mean: P-value=0.010, F(1.4,30.3)=6.3; T-mean: P-value=0.032, F(1.4,29.2)=4.5). However, Bonferroni post-hoc tests showed no significant differences between the trials for F-peak and T-mean (P-value>0.05). Although, trial 1 was significantly different from trial 2 and trial 3 in Bonferroni Post Hoc tests of F-mean (P-value<0.05, Figure 4).

The dominant leg was not significantly different compared to the non-dominant leg (F-peak: P-value=0.071, F(1,21)=3.6; T-peak: P-value=0.114, F(1,21)=2.7; F-mean: P-value=0.058, F(1,21)=4.0; T-mean: P-value=0.098, F(1,21)=3.0). Although for all outcomes there was a measurement*leg interaction effect (P-value<0.05, F(1,21)=11.2, 9.7, 10.9, 9.7 for respectively F-peak, F-mean, T-peak, and T-mean). The interaction effects measurement*trial, trial*leg and measurement*trial*leg were not significant (P-value>0.05).


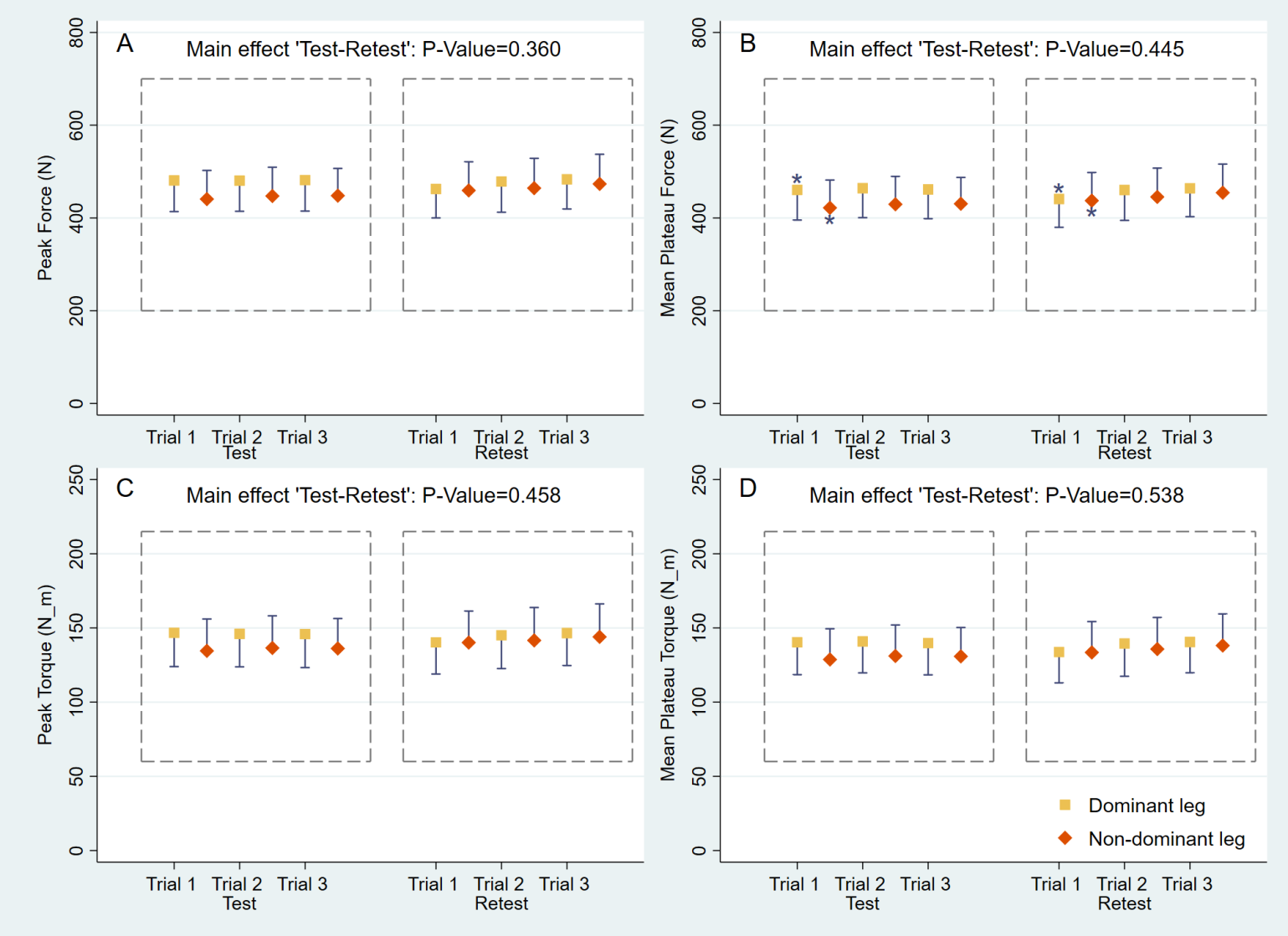


Figure S2.1: Mean with 95% confidence interval (plotted to one side) of the three trials at the Q-Force ӀӀ of the test and retest for the dominant and non-dominant leg (n=22). A) Peak force; B) The average force of the plateau phase; C) Peak Torque; D) The average torque of the plateau phase. *Trial 1 is significant different from trial 2 and 3 in Bonferroni post hoc tests (P-value<0.05).

Table S2.2: The Intra Class Correlation between the trial 1 & 2, 1 & 3, and 2 &3 for the test and re-test test for the peak torque and the mean torque of the plateau phase for the dominant and non-dominant leg (n=22).

|  | **Peak force (N)** | | | | **Mean force (N)** | | | |
| --- | --- | --- | --- | --- | --- | --- | --- | --- |
|  | **Dominant leg** | | **Non-dominant leg** | | **Dominant leg** | | **Non-dominant leg** | |
| **Test** | **Trial 2** | **Trial 3** | **Trial 2** | **Trial 3** | **Trial 2** | **Trial 3** | **Trial 2** | **Trial 3** |
| **Trial 1** | 0.985 | 0.975 | 0.971 | 0.966 | 0.986 | 0.964 | 0.959 | 0.961 |
| **Trial 2** | X | 0.985 | X | 0.968 | X | 0.980 | X | 0.966 |
| **Re-test** |  |  |  |  |  |  |  |  |
| **Trial 1** | 0.972 | 0.943 | 0.978 | 0.969 | 0.962 | 0.931 | 0.987 | 0.975 |
| **Trial 2** | X | 0.979 | X | 0.992 | X | 0.984 | X | 0.992 |
|  | **Peak Torque (Nm)** | | | | **Mean Torque (Nm)** | | | |
|  | **Dominant leg** | | **Non-dominant leg** | | **Dominant leg** | | **Non-dominant leg** | |
| **Test** | **Trial 2** | **Trial 3** | **Trial 2** | **Trial 3** | **Trial 2** | **Trial 3** | **Trial 2** | **Trial 3** |
| **Trial 1** | 0.987 | 0.977 | 0.977 | 0.972 | 0.986 | 0.966 | 0.967 | 0.968 |
| **Trial 2** | X | 0.987 | X | 0.973 | X | 0.983 | X | 0.971 |
| **Re-test** |  |  |  |  |  |  |  |  |
| **Trial 1** | 0.987 | 0.953 | 0.983 | 0.975 | 0.971 | 0.945 | 0.991 | 0.980 |
| **Trial 2** | X | 0.981 | X | 0.994 | X | 0.985 | X | 0.994 |

For all values P-value < 0.001

Table S2.3: The Intra Class Correlation between the three trials for the test and re-test test for the peak torque and the mean torque of the plateau phase for the dominant and non-dominant leg (n=22).

| **Force (N)** | **Leg** | **Test**  **Trial 1-3** | **Retest**  **Trail 1-3** |
| --- | --- | --- | --- |
| **Peak** | D | 0.982 | 0.965 |
|  | ND | 0.969 | 0.980 |
| **Mean** | D | 0.977 | 0.959 |
|  | ND | 0.962 | 0.985 |
| **Torque (Nm)** |  |  |  |
| **Peak** | D | 0.983 | 0.971 |
|  | ND | 0.974 | 0.984 |
| **Mean** | D | 0.987 | 0.967 |
|  | ND | 0.969 | 0.988 |

For all values P-value < 0.001

## LIST OF ABBREVIATIONS

F-mean – The average of the force of the plateau phase

F-peak – Peak force, defined as the maximum value during a trial

ICC – Intra class correlation

IQR – Interquartile range

MDC – Minimal Detectable Change

SEM – Standard Error of Measurement

T-mean – The average of the torque of the plateau phase

T-peak – Peak torque, defined as the maximum value during a trial
